# Rubesanolides F and G: Two Novel Lactone-Type Norditerpenoids from *Isodon rubescens*

**DOI:** 10.3390/molecules26133865

**Published:** 2021-06-24

**Authors:** Kang He, Juan Zou, Yu-Xue Wang, Chen-Liang Zhao, Jiang-Hai Ye, Jing-Jie Zhang, Lu-Tai Pan, Hong-Jie Zhang

**Affiliations:** 1 The Key Laboratory of Miao Medicine of Guizhou Province, Guizhou University of Traditional Chinese Medicine, Guiyang 550025, China; hekang0851@163.com (K.H.); zoujuan466@gzy.edu.cn (J.Z.); wyx893849828@sina.com (Y.-X.W.); 18482082@life.hkbu.edu.hk (C.-L.Z.); yjh20160601@163.com (J.-H.Y.); zjj523@126.com (J.-J.Z.); 2 School of Chinese Medicine, Hong Kong Baptist University, Kowloon, Hong Kong, China

**Keywords:** *Isodon rubescens*, norditerpenoids, ECD calculation, single-crystal X-ray diffraction, biological activities

## Abstract

A phytochemical investigation of the leaves of the medicinal plant *Isodon rubescens* led to the isolation of the two new degraded abietane lactone diterpenoids rubesanolides F (**1**) and G (**2**). Their structures were elucidated based on the analyses of the HRESIMS and 1D/2D NMR spectral data, and their absolute configurations were determined by ECD spectrum calculations and X-ray single crystal diffraction methods. Compounds **1** and **2**, with a unique γ-lactone subgroup between C-8 and C-20, were found to form a carbonyl carbon at C-13 by removal of the isopropyl group in an abietane diterpene skeleton. Rubesanolide G (**2**) is a rare case of abietane that possesses a *cis*-fused configuration between rings B and C. The two isolates were evaluated for their biological activities against two cancer cell lines (A549 and HL60), three fungal strains (*Candida alba*, *Aspergillus niger* and *Rhizopus nigricans*) and three bacterial strains (*Escherichia coli*, *Staphylococcus aureus* and *Bacillus subtilis*).

## 1. Introduction

*Isodon*, a genus belonging to Labiatae family, contains about 150 plant species [1]. The genus has attracted considerable attention as a prolific source of diterpenoids with diverse structures and biological properties [2]. More than 50 *Isodon* species have been phytochemically investigated, leading to isolation of a large number of different types of diterpenoids, including kauranes, abietanes, labdanes, pimaranes, isopimaranes, gibberellanes, clerodanes and atisanes [3,4,5]. However, the lactone-type norditerpenoids, formed by losing the isopropyl group in C-ring of an abietane skeleton, has not been reported previously.

**Figure 1:**
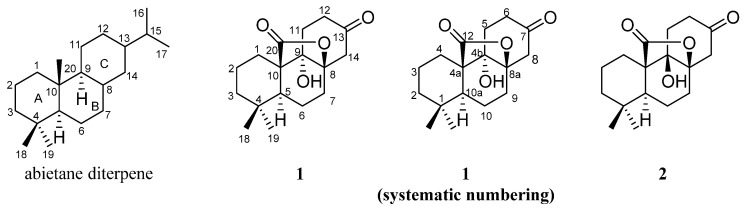
The structures of abietane diterpene and compounds **1** and **2**.

*Isodon rubescens* (Hemsl.) Hara, a well-known folk medicine found primarily in China, has been used for treatment of respiratory and gastrointestinal bacterial infections, inflammation and cancer [6]. About 200 new diterpenoids, including several with novel skeletons such as dimeric *ent*-kauranoids, have been reported from this species through phytochemical isolation of over seven samples collected in different regions of China [7]. Our previous investigations on this plant discovered several novel diterpenoids (rubesanolides A–E), which were found to contain an unprecedented β-lactone group formed between C-9 and C-20 for rubesanolide A and a unique γ-lactone subgroup formed between C-8 and C-20 for rubesanolides C-E [8,9]. In our continuing efforts to discover new diterpene lead molecules with diverse structures and bioactivities, we have reinvestigated the leaves of *I. rubescens*. As a result, we have identified two norditerpenes[rubesanolides F (**1**) and G (**2**)] (Figure 1), which possess a unique skeleton containing only 17 carbons. The skeleton is formed with a carbonyl carbon at C-13 through degradation of the isopropyl group in the ring-C of an abietane skeleton. Interestingly, rubesanolide G (**2**) is shown to be a rare case for possessing a *cis*-fused six-membered ring between rings B and C, which is contrary to the *trans*-fused conformation normally formed between rings B and C in an abietane diterpene. This is the first time that a pair of epimers of degraded lactone-type abietane diterpenes have been discovered in the plants of the *Isodon* genus. We herein report their structural identification, proposed biosynthetic pathways as well as their biological activity evaluation against cancer cell lines, bacterial strains and fungal strains.

## 2. Results and Discussion

### 2.1. Structure Elucidation of Compounds

Compound **1** was obtained as colorless cubic crystals (MeOH). Its molecular formula, C_17_H_24_O_4_, determined by means of analyzing its NMR and HRESIMS [*m/z* 315.1564 [M + H]^+^ (calcd 315.1567)] data, was calculated to have six degrees of unsaturations. The IR spectrum of **1** showed the absorptions of hydroxyl (3339 cm^−1^), ketone (1709 cm^−1^) and lactone (1759 cm^−1^) groups. In the ^1^H and ^13^C-NMR spectra (Table 1), **1** was found to have 17 carbon resonances due to six quaternary carbons, one tertiary carbon, eight methylenes, and two methyl groups. The 17 carbons were characterized as a methine group ([*δ*_H_ 2.29 (dd, *J* = 13.0, 5.6 Hz, H-5); *δ*_C_ 42.0 (d, C-5)], two tertiary methyl groups [*δ*_H_ 0.87 (s, Me-18) and 1.02 (s, Me-19)], two carbonyl groups [*δ*_C_ 179.0 (s, C-20), and 207.4 (s, C-13)], two oxyquaternary carbons [*δ*_C_ 87.9 (s, C-8), and 76.2 (s, C-9)], and two quaternary carbons [*δ*_C_ 33.9 (s, C-4) and 53.6 (s, C-10)]. By comparing to the NMR and MS data of the known rubesanolide E, compound **1** was determined to be an abietane diterpenoid with the isopropyl group being degraded.

**Figure 2:**
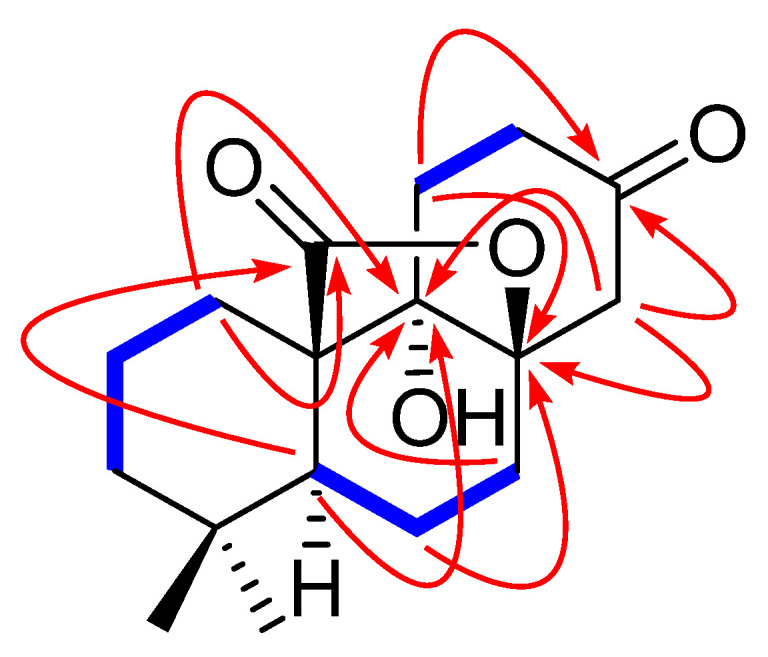
Key ^1^H-^1^H COSY (blue and bold) and HMBC (red arrows) correlations of **1**.

The presence of the HMBC correlations (Figure 2) from the proton signals at *δ*_H_ 1.41 (H-1), 2.29 (H-5), 1.64 (H-7), 2.34 and 3.00 (H-12), 2.80 (H-14) to C-9 (*δ*_C_ 76.2) determined a hydroxyl group at C-9. The ketone group belonging to C-13 (*δ*_C_ 207.4) was deduced from the observation of the HMBC correlations of H-11 (*δ*_H_ 2.01 and 2.20), H-12 (*δ*_H_ 2.34 and 3.00) and H-14 (*δ*_H_ 2.80 and 2.82) to C-13. In addition, besides the five degrees of unsaturations ascribed to two carbonyl groups and three six-membered rings, the remaining unsaturation required compound **1** to contain a cyclic ring system, which is assigned as a γ-lactone moiety formed at C-8 and C-10 positions due to the ^13^C-NMR [*δ*_C_ 87.9 (C-8), 76.2 (C-9), 53.6 (C-10) and 179.0 (C-20)] and the presence of the HMBC correlations of H-1 (*δ*_H_ 1.41 and 1.90) and H-5 to C-20 and H-6 [*δ*_H_ 1.75 (dd, *J* = 13.7, 4.7 Hz)], H-11 (*δ*_H_ 2.20) and H-14 (*δ*_H_ 2.80) to C-8.

**Figure 3:**
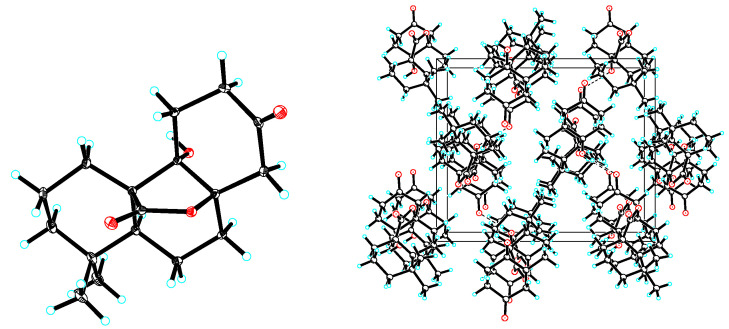
X-ray crystallographic structure of **1**.

**Figure 4:**
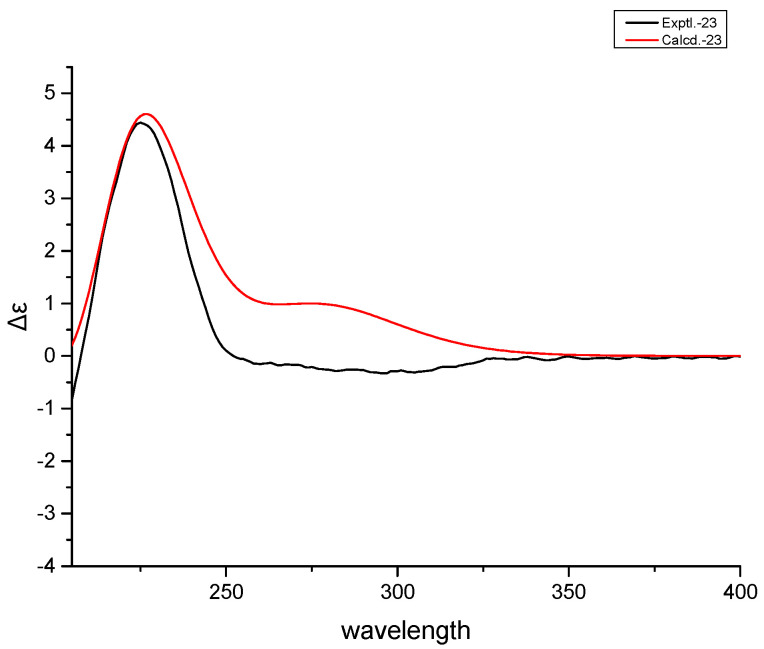
Experimental (black) and calculated ECD (red) spectra of **1**.

To determine the stereochemistry and the absolute configuration, **1** was crystallized in MeOH to afford a colorless crystal of the orthorhombic space group P212121, which was analyzed by X-ray crystallography (Figure 3). The absolute configuration of **1** was determined by structural refinement through the measurement of the Flack parameter [10]. In our study, the final refinement on the Cu Kα data of the crystal of **1** resulted in a Flack parameter of 0.04 (3), allowing an unambiguous assignment of the absolute configuration of the chiral centers as shown in Figure 1. The four chiral centers: C-5, C-8, C-9 and C-10, were thus determined as *S*, *R*, *S* and *R* respectively. To further validate the determined absolute stereochemistry of **1**, the electronic circular dichroism (ECD) calculation of **1** was performed. As shown in Figure 4, the calculated ECD curve of **1** matched closely to the experimental data, which confirmed the absolute configurations of the four chiral centers in the structure of **1** as 5*S*, 8*R*, 9*S* and 10*R*. Accordingly, **1** was established as (4a*R*,4b*S*,8a*R*,10a*S*)-4b-hydroxy-1,1-dimethyloctahydro-2*H*,6*H*-8a,4a-(epoxymethano)phenanthrene-7,12(8*H*)-dione and given the trivial name rubesanolide F.

**Figure 5:**
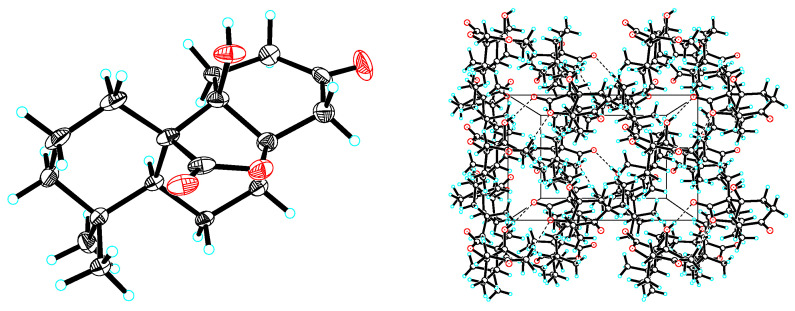
X-ray crystallographic structure of **2**.

Compound **2** was obtained as colorless cubic crystals (MeOH) with the molecular formula determined as C_17_H_24_O_4_ through the analysis of its NMR and HRESIMS data [*m/z* 315.1564 [M + Na]^+^ (calculated as 315.1567)]. The IR spectrum of **2** displayed the absorptions of hydroxyl (3452 cm^−1^), carbonyl (1693 cm^−1^) and lactone (1719 cm^−1^) groups. The similarity of the NMR spectroscopic data and the same molecular formula of **2** to **1** suggested that the two compounds possessed the same planar structure. The significant differences of the ^13^C-NMR data at C-8 (*δ*_C_ 84.3), C-9 (*δ*_C_ 80.5) and C-11 (*δ*_C_ 24.4) indicated that **2** differs from **1** only by the stereochemistry at C-9, which was confirmed by the X-ray crystallographic analysis of a crystal obtained from the crystallization of **2** in MeOH (Figure 5).

**Figure 6:**
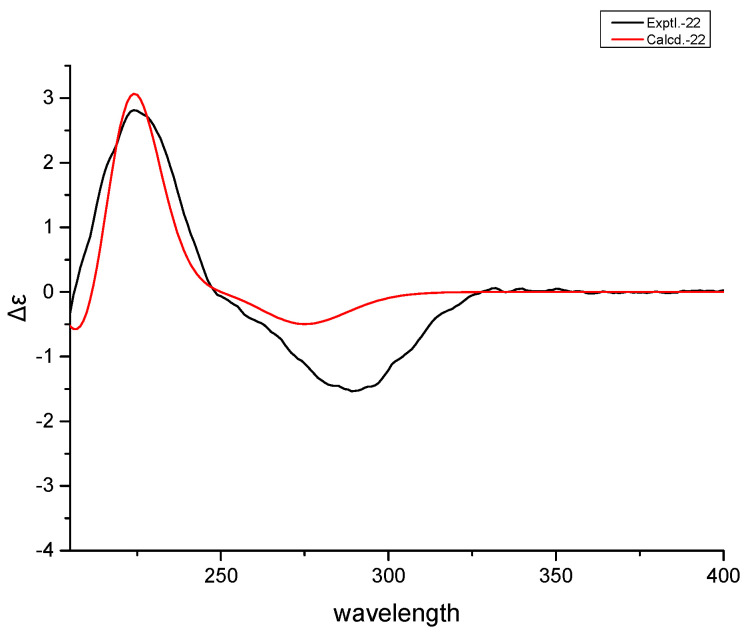
Experimental (black) and calculated ECD (red) spectra of **2**.

The crystal possessed the orthorhombic space group P212121 and was found to have a Flack parameter of 0.17 (5) through the refinement on the Cu Kα data, which assigned the absolute structure of **2** as shown in Figure 1. The four chiral centers of C-5, C-8, C-9 and C-10 were thus determined as *S*, *R*, *R* and *R*, respectively. The calculated ECD curve of **2** that closely matches the experimental data further validated the assignment of the absolute configurations of the four chiral centers (Figure 6). Accordingly, the structure of **2** was established as (4a*R*,4b*R*,8a*R*,10a*S*)-4b-hydroxy-1,1-dimethyloctahydro-2*H*,6*H*-8a,4a-(epoxymethano)phenanthrene-7,12(8*H*)-dione and given the trivial name rubesanolide G.

### 2.2. Hypothetical Biosynthetic Pathways of ***1*** and ***2***

**Scheme 1:**
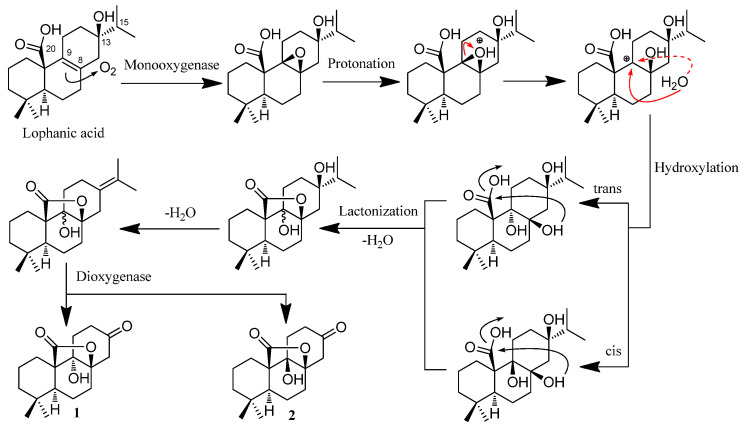
Proposed biogenetic pathways of **1** and **2**.

Rubesanolides F and G (**1** and **2**) are two unique lactone-type norditerpenoids that were determined to possess only 17 carbons in their degraded tricyclohexane abietane skeleton. They could be formed from lophanic acid through the plausible biogenetic pathway depicted in Scheme 1. Lophanic acid was found to be a major component (0.46%) in an *Isodon* species (*I. lophanthoides*) [11,12] and is therefore considered to be a plausible biosynthetic precursor of the two norditerpenoids. The monooxygenase enzyme converts the carbon–carbon double bond between C-8 and C-9 in lophanic acid to an epoxide, which can be converted to two *trans*-dihydroxyl groups and two *cis*-dihydroxyl groups through forming a carbon cation at C-9 as an intermediate. Lactonization of the hydroxyl group at C-8 with the C-20 carboxylic acid group yields the γ-lactone group formed between C-8 and C-20. Dehydration of the hydroxy group of C-13 produces the double bond between C-13 and C-15, which is cleaved by a dioxygenase enzyme to form the ketone group at C-13. 

### 2.3. Bioactivity Evaluation Against Cancer Cell Lines and Fungal and Bacterial Strains

Compounds **1** and **2** were evaluated for their cytotoxicity against the A549 (lung) and HL60 (leukemia) cell lines, antimicrobial effects against the fungal strains of *Candida alba*, *Aspergillus niger* and *Rhizopus nigricans* and the bacterial strains of *Escherichia coli*, *Staphylococcus aureus* and *Bacillus subtilis*. In the assay tests, the compounds **1** and **2** showed only slight activity effects against A549 with inhibitory rates of 15.9% and 11.7%, respectively, at the concentration of 10 μM. However, no cytotoxicity against HL-60 cell line at 10 μM and no inhibitory effects against the tested three fungi and three bacteria at 100 μM were observed for the two compounds.

## 3. Materials and Methods

### 3.1. General Experimental Procedures

Optical rotation was measured with a Jasco P-1010 polarimeter (Perkin-Elmer, Waltham, United States). Melting points were obtained on a XRC-1 micro melting point apparatus (Sichuan Weitai optical instrument factory, Chengdu, China). UV spectra were obtained using a Shimadzu UV-2401A spectrophotometer (Shimadzu, Kyoto, Japan). A VECTOR22 spectrophotometer (Bruker, Rheinstetten, Germany) with KBr pellets was used for scanning IR spectroscopy. 1D and 2D NMR spectra were recorded on a Bruker Avance 600 MHz spectrometer (Bruker, Rheinstetten, Germany). Chemical shifts (*δ*) were expressed in ppm with reference to the solvent signals. High-resolution electrospray ionization mass spectroscopy (HR-ESI-MS) was performed on a Bruker Q-TOF spectrometer (Bruker, Rheinstetten, Germany). X-ray crystallographic data were obtained on a Bruker APEX-II CCD instrument using Cu Kα radiation (Bruker, Rheinstetten, Germany). Column chromatography was performed with silica gel (200–300 mesh; Qingdao Marine Chemical, Inc., Qingdao, China). Thin-layer chromatography (TLC) was carried out on silica gel GF_254_ coated on glass plates (Qingdao Marine Chemical Inc., Qingdao, China) using various solvent systems and spots were visualized by heating the silica gel plates sprayed with 95–98% H_2_SO_4_-EtOH (*V*/*V* = 10:90). All solvents including petroleum ether (60–90 °C) were distilled prior to use.

### 3.2. Plant Material

The plant materials (stems and leaves) of *Isodon rubesens* were collected in Guiyang, Guizhou Province, China, in July 2018. The voucher specimen was identified by Professor Junhua Zhao of Guizhou University of Traditional Chinese Medicine and deposited at Guizhou University of Traditional Chinese Medicine with the number of the voucher specimen as No.20180713.

### 3.3. Extraction and Isolation

The air-dried and powdered leaves of *I. rubescens* (8.8 kg) were percolated with MeOH at room temperature (3 × 10 L). All the extracts were combined and concentrated under vacuum to give a residue (1.3 kg), which was suspended in water and sequentially extracted with petroleum ether (1 L × 2) and EtOAc (1 L × 3). The EtOAc part (230.2 g) was chromatographed on a silica gel column eluted with petroleum ether/EtOAc (100:1–0:1) to furnish 6 fractions (A–F). Fraction C (37.4 g) was subjected to a RP-8 (octylsilane bonded silica gel) column with a gradient eluent of MeOH/H_2_O (5:95–50:50) to afford fractions C1-C4. Fraction C2 (6.8 g) was subjected to another silica gel column eluting with gradient CH_2_Cl_2_/EtOAc solvent system (100:1–1:1) to afford compounds **1** (15 mg) and **2** (17 mg), which were then recrystallized by a slow evaporation from MeOH at room temperature to provide their respective colorless crystals suitable for single-crystal X-ray diffraction analysis.

### 3.4. Characerization of Compounds ***1*** and ***2***

Compound **1**. Colorless crystals (MeOH); mp 256.4–257.6 °C; HR-ESI-MS ([M + Na]^+^ *m*/*z* 315.1564, calcd 315.1567 for C_17_H_24_O_4_). ${\left[\alpha \right]}_{D}^{22}$ +68.97 (*c* 1.74, pyridine); UV (pyridine) *λ*_max_ (log ε) 304 (0.19) nm; IR (KBr) *ν*_max_ 3339, 2962, 2932, 1759, 1709, 1459, 1366, 1322, 1224, 1174, 1125, 1091, 1044, 1016, 949 cm^−1^; ^1^H and ^13^C-NMR data, see Table 1.

Compound **2**. Colorless crystals (MeOH); mp 245.5–246.5 °C; HR-ESI-MS ([M + Na]^+^ *m*/*z* 315.1564, calcd 315.1567 for C_17_H_24_O_4_). ${\left[\alpha \right]}_{D}^{28.6}$ +8.62 (c 0.20, MeOH); UV (CHCl_3_) *λ*_max_ (log ε) 242 (0.24) nm; IR (KBr) *ν*_max_ 3452, 2953, 1769, 1719, 1693, 1454, 1368, 1226, 1184, 1119, 1070, 1002, 956, 865 cm^−1^; ^1^H and ^13^C-NMR data, see Table 1.

### 3.5. Theoretically Calculated ECD Spectra of ***1*** and ***2***

The 3D structures were obtained from X-ray data, and the ECD calculations were performed using the TDDFT methodology at b3lyp/6-311+g(d,p) level in methanol. The ECD spectra were simulated by the SpecDis program (University of Würzburg, Würzburg, Germany).

### 3.6. X-ray Crystallographic Analysis

Colorless cubic crystals of compounds **1** and **2** were obtained from methanol. The X-ray diffraction data were collected on a Bruker APEX-II CCD instrument using Cu Kα radiation (λ = 1.54184 Å) for **1** and **2**. The molecule structures of the two compounds were built up from tricyclohexane system bearing *δ*-lactone, methyl, hydroxyl group and carbonyl group substituents. The X-ray analysis determined the absolute configurations. In the molecule of **1**, H-5 and the C-9 hydroxyl group are *α* oriented, whereas the *δ*-lactone ring between C-8 and C-9 is *β* oriented. The only difference between **2** and **1** is that the C-9 hydroxyl group in **2** is *β* oriented. In the skeleton of **1**, the three *trans*-fused six-membered rings A [(atoms C-1 to C-10), B (atoms C-5 to C-10) and C (atoms C-8 to C-14)] adopt chair conformations. In the skeleton of **2**, the *trans*-fused six-membered rings A and B adopt chair conformations, but rings B and C are *cis*-fused, which adopt chair and half-chair conformations, respectively.

### 3.7. Bioactivity Evaluation

Compounds **1** and **2** were evaluated for their biological activities against various strains of pathogenic fungi and bacteria and two human cancer cell lines.

Cytotoxicity assays involving human non-small cell lung cancer (A549) and human leukemia cells (HL60) were performed in quintuplicate in 96-well microplates (Corning Inc., Corning, NY, USA), and the viable cells at the end of the incubation period were quantified using MTT as previously reported [13]. The A549 and HL60 cells were cultured in RPMI 1640 medium supplemented with 10% fetal bovine serum (10% FBS, Gibco, Grand Island, NY, USA). Cells were incubated at 37 °C with 5% CO_2_ in a humidified atmosphere. All compounds were dissolved in DMSO to make a stock solution. Adriamycin was used as positive control. Each sample was tested three times independently and the results are expressed as M ± SD.

The bacteriostatic activities of compounds **1** and **2** were evaluated using the agar plate punch assay [14]. All tested samples were dissolved in DMSO at a concentration of 100 μM. Then, 40 μL of each of the sample solution was added onto a well (6.18 mm in diameter) that had been punched in the appropriate agar growth medium smeared with a suspension of the test organism. Gentamicin (Sigma Chemical Co, St. Louis, MO, USA) was used as the standard antibiotics for *E. coli*, *S. aureus* and *B. subtilis*. Nystatin (Sigma Chemical Co, St. Louis, USA) was used as positive control against *C. alba*, *A. niger* and *R. nigricans*. When the measurement diameter of the inhibition by a compound (measured by Vernier caliper) is greater than 10 mm, the compound would be selected to obtain its MIC value. Each sample was tested for three times independently and the results are expressed as M ± SD.

## 4. Conclusions

In summary, we have identified two new degraded lactone-type abietane diterpenoids [rubesanolides F (**1**) and G (**2**)]. The structures were elucidated by NMR and MS spectroscopic data, and their absolute configurations were confirmed by ECD spectral data and by X-ray crystallographic analysis. The two compounds were determined to belong to a degraded abietane diterpenoids with only 17 carbons in the skeleton. The unique nor-abietanes are found with a γ-lactone subgroup between C-8 and C-20 and they lack an isopropyl group that is commonly found in an abietane diterpenoid. The structure’s uniqueness is further demonstrated in rubesanolide G (**2**). The compound possesses a pair of *cis*-fused six member rings between rings B and C, which is strikingly in contrast to the common abietane structure having a pair of *trans*-fused B and C rings. This represents the first discovery for the natural degraded lactone-type abietane diterpenes. The two compounds showed weak or no bioactivities in our screened cytotoxicity and antimicrobial assays. However, the structure novelty of the compounds shows them to be lead molecules, which may be explored as synthetic scaffolds to prepare a compound library for drugs for further bioactivity studies.

## Figures and Tables

**Table 1:** ^1^H and ^13^C-NMR spectral data of **1** and **2**.

No.	1 ^a^		2 ^b^	
	*δ*_H_*,* (*J* in Hz) ^c^	*δ*_C_	*δ*_H_*,* (*J* in Hz) ^c^	*δ*_C_
1a	1.41 (m)	26.0 t	1.21 (brtd, 13.9, 5.0)	25.2 t
1b	1.90 (d, 13.1)		2.11 (m)	
2a	1.47 (m)	19.0 t	1.58 (m)	18.0 t
2b	2.26 (m)		2.08 (m)	
3a	1.15 (brt, 13.4)	42.1 t	1.11 (brtd, 13.5, 3.0)	41.3 t
3b	1.39 (m)		1.48 (m)	
4	–	33.9 s	–	33.8 s
5	2.29 (dd, 13.0, 5.6)	42.0 d	1.41 (dd, 13.4, 4.7)	44.4 d
6a	1.47 (m)	20.8 t	1.50 (m)	19.8 t
6b	1.75 (dd, 13.7, 4.7)		1.81 (m)	
7a	1.64 (dd, 13.3, 5.2)	30.9 t	1.62 (m)	31.6 t
7b	2.42 (dd, 13.4, 9.1)		1.91 (dd, 15.5, 7.2)	
8	–	87.9 s	–	84.3 s
9	–	76.2 s	–	80.5 s
10	–	53.6 s	–	52.8 s
11a	2.01 (brtd, 14.6, 2.0)	32.4 t	1.86 (d, 5.7)	24.4 t
11b	2.20 (d, 14.5)		2.15 (ddd, 13.7, 11.4, 6.2)	
12a	2.34 (dd, 13.8, 2.1)	37.0 t	2.44 (dd, 16.6, 6.2)	36.2 t
12b	3.00 (m)		2.75 (m)	
13	–	207.4 s	–	207.2 s
14a	2.82 (d, 14.3)	46.4 t	2.47 (d, 14.4)	47.0 t
14b	2.80 (d, 14.3)		3.28 (d, 14.4)	
18	0.87 (s)	32.4 q	0.89 (s)	32.2 q
19	1.02 (s)	20.8 q	0.90 (s)	20.0 q
20	–	179.0 s	–	179.3 s

Data (^a^ C_5_D_5_N and ^b^ CDCl_3_) were recorded on a Bruker Avance 600 MHz spectrometer. ^c^ Multiplicities in parentheses represent: s (singlet), dd (doublet of doublet), td (triplet of doublet) and m (multiplet).

## Data Availability

The data presented in this study are available in article and Supplementary Material.

## Supplementary Materials

The following are available online. Spectra S1: ^1^H-NMR spectrum of rubesanolide F (**1**), Spectra S2: ^13^C-NMR and DEPT of rubesanolide F (**1**), Spectra S3: HSQC spectrum of rubesanolide F (**1**), Spectra S4: HMBC spectrum of rubesanolide F (**1**), Spectra S5: COSY spectrum of rubesanolide F (**1**), Spectra S6: ROESY spectrum of rubesanolide F (**1**), Spectra S7: HRESIMS spectrum of rubesanolide F (**1**), Spectra S8: The IR spectrum of rubesanolides F (**1**), Spectra S9: ^1^H-NMR spectrum of rubesanolide G (**2**), Spectra S10: ^13^C-NMR and DEPT spectra of rubesanolide G (**2**), Spectra S11: HSQC spectrum of rubesanolide G (**2**), Spectra S12: HMBC spectrum of rubesanolide G (**2**), Spectra S13: COSY spectrum of rubesanolide G (**2**), Spectra S14: ROESY spectrum of rubesanolide G (**2**), Spectra 15: HRESIMS data of rubesanolide G (**2**), Spectra 16: The IR spectrum of rubesanolide G (**2**).

## Sample Availability

Samples of the compounds **1** and **2** are available from the authors.
