# Supplementary material for: Rubesanolides F and G: Two Novel Lactone-Type Norditerpenoids from Isodon rubescens
Source: Molecules. 2021 Jun 24;26(13):3865. doi: 10.3390/molecules26133865 (PMC8270274; doi:10.3390/molecules26133865)
Supplement: Supplementary file 1 [file molecules-26-03865-s001.zip › molecules-1269360-supplementary.pdf]

Supplementary Materials

Rubesanolides F and G: two novel lactone-type norditerpenoids from *Isodon rubescens*

[1] The Key Laboratory of Miao Medicine of Guizhou Province, Guizhou University of Traditional Chinese Medicine, Guiyang 550025, China; hekang0851@163.com (K.H.); zoujuan466@gzy.edu.cn (J.Z.); wyx893849828@sina.com (Y.-X.W.); 18482082@life.hkbu.edu.hk (C.-L.Z.); yjh20160601@163.com (J.-H.Y.); zjj523@126.com (J.-J.Z.)

[2] School of Chinese Medicine, Hong Kong Baptist University, Kowloon, Hong Kong, China

* Correspondence: ltpan@sina.cn (L.-T.P.), zhanghj@hkbu.edu.hk (H.-J.Z.)

† These authors contributed equally to this work and are co-first authors

Supporting Information List

Characterization Data of New Compounds **1** and **2**

► ^{1}H−NMR, ^{13}C-NMR, DEPT, HSQC, HMBC, COSY, NOESY HRESIMS and IR spectra of rubesanolide F (**1**)

► ^{1}H−NMR, ^{13}C-NMR, DEPT, HSQC, HMBC, COSY, NOESY HRESIMS and IR spectra of rubesanolide G (**2**)

For rubesanolide F (1):

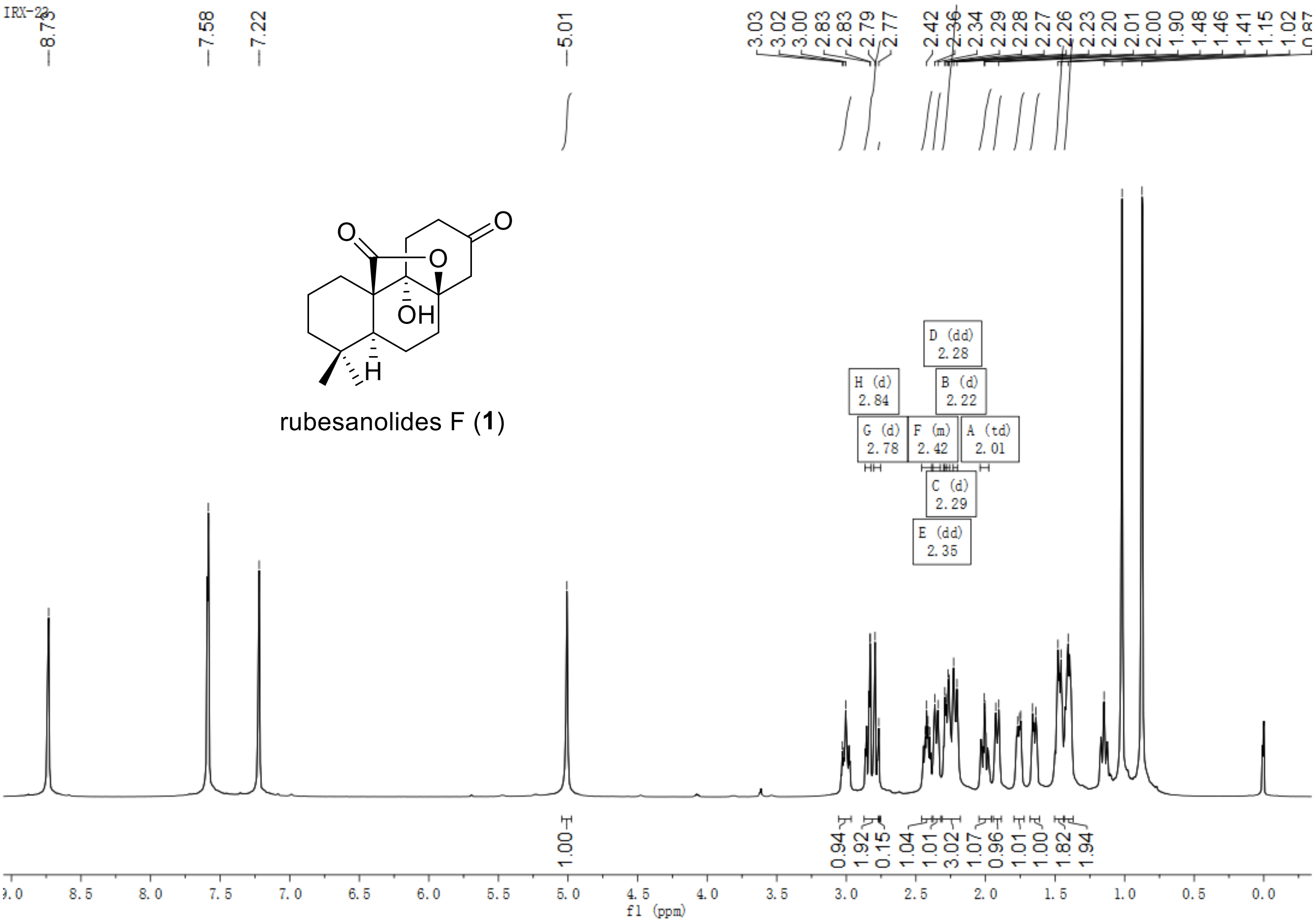

Spectra 1. ^{1}H-NMR spectrum of rubesanolide F (**1**)

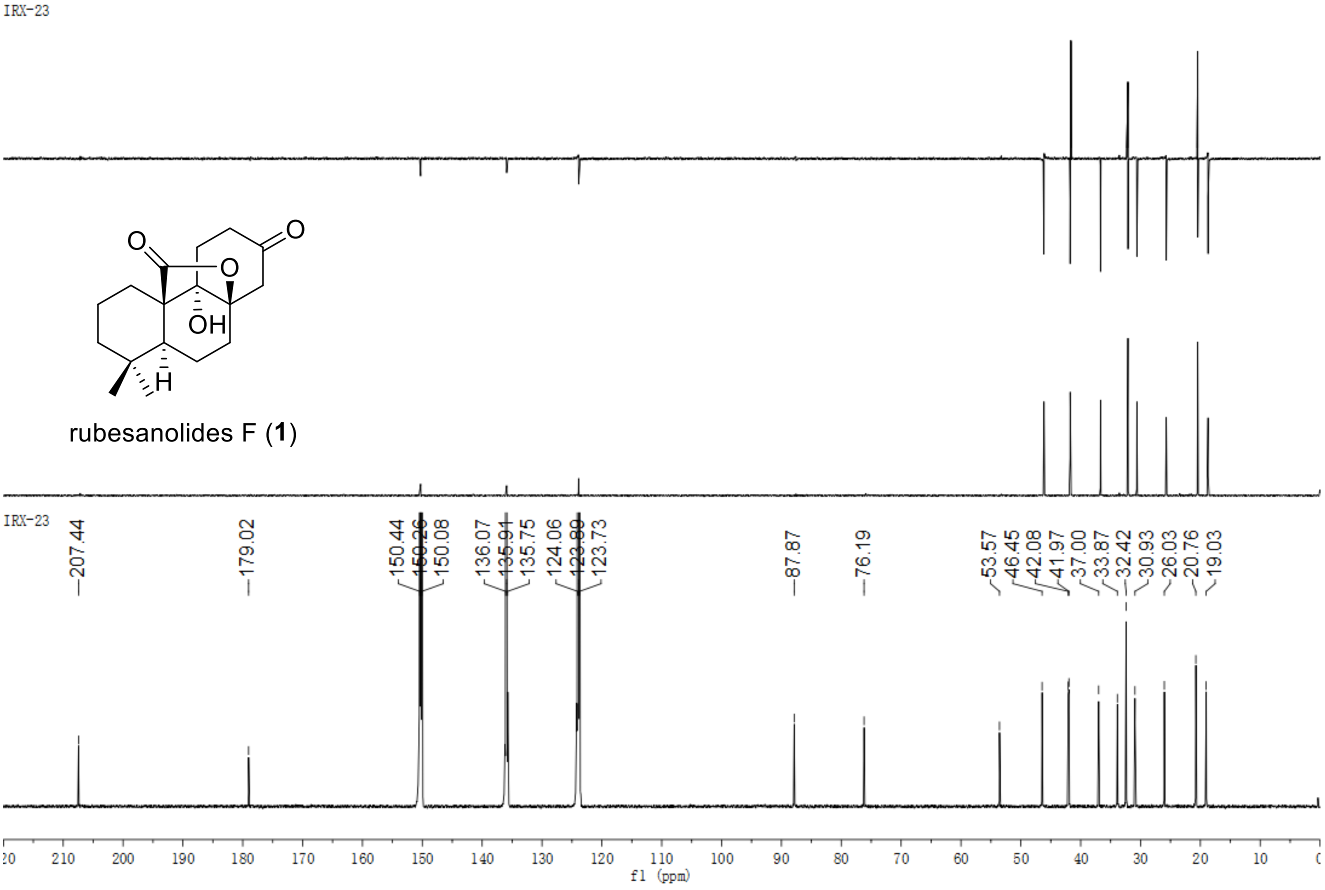

Spectra 2. ^{13}C-NMR and DEPT of rubesanolide F (**1**)

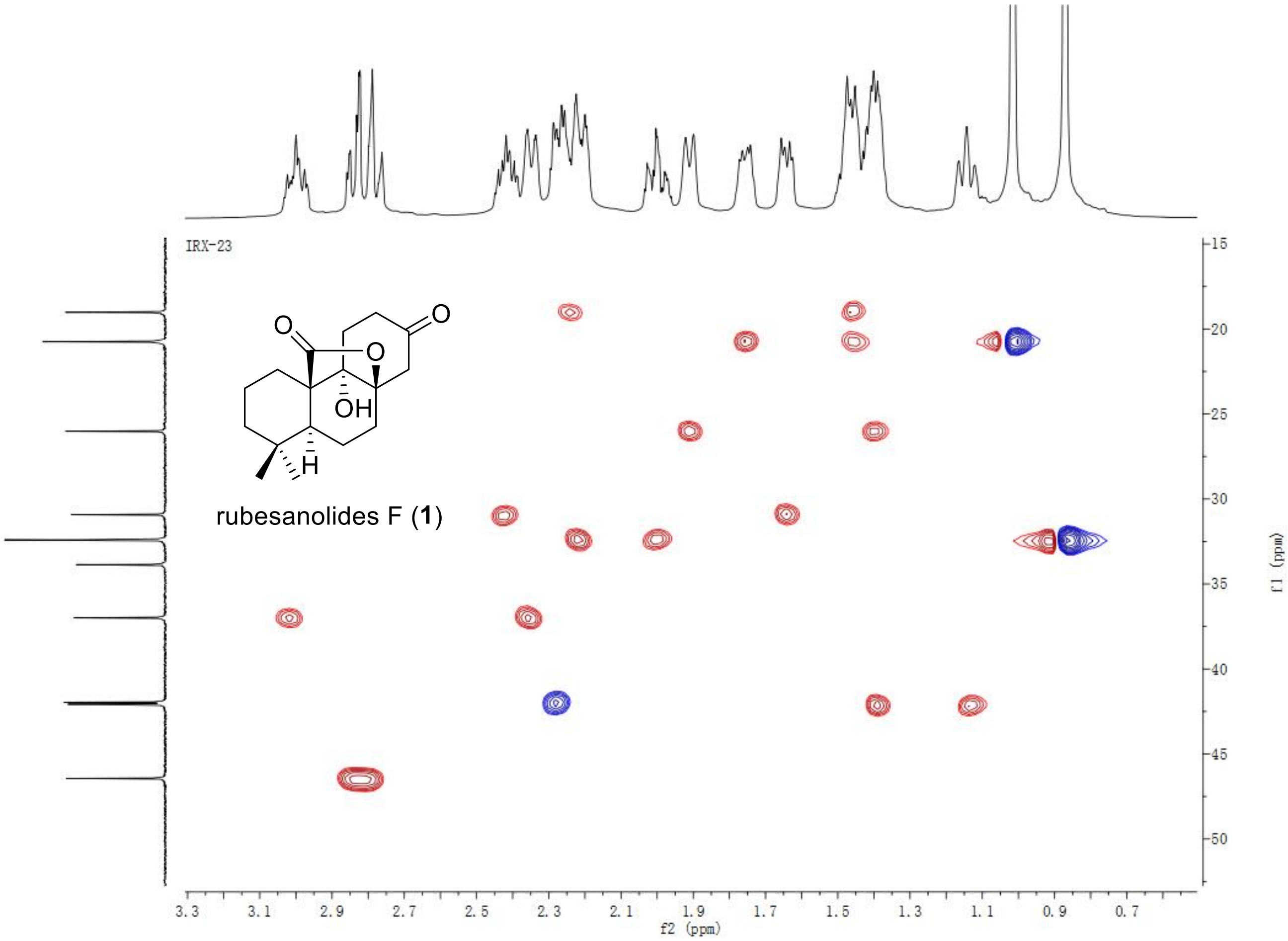

Spectra 3. HSQC spectrum of rubesanolide F (**1**)

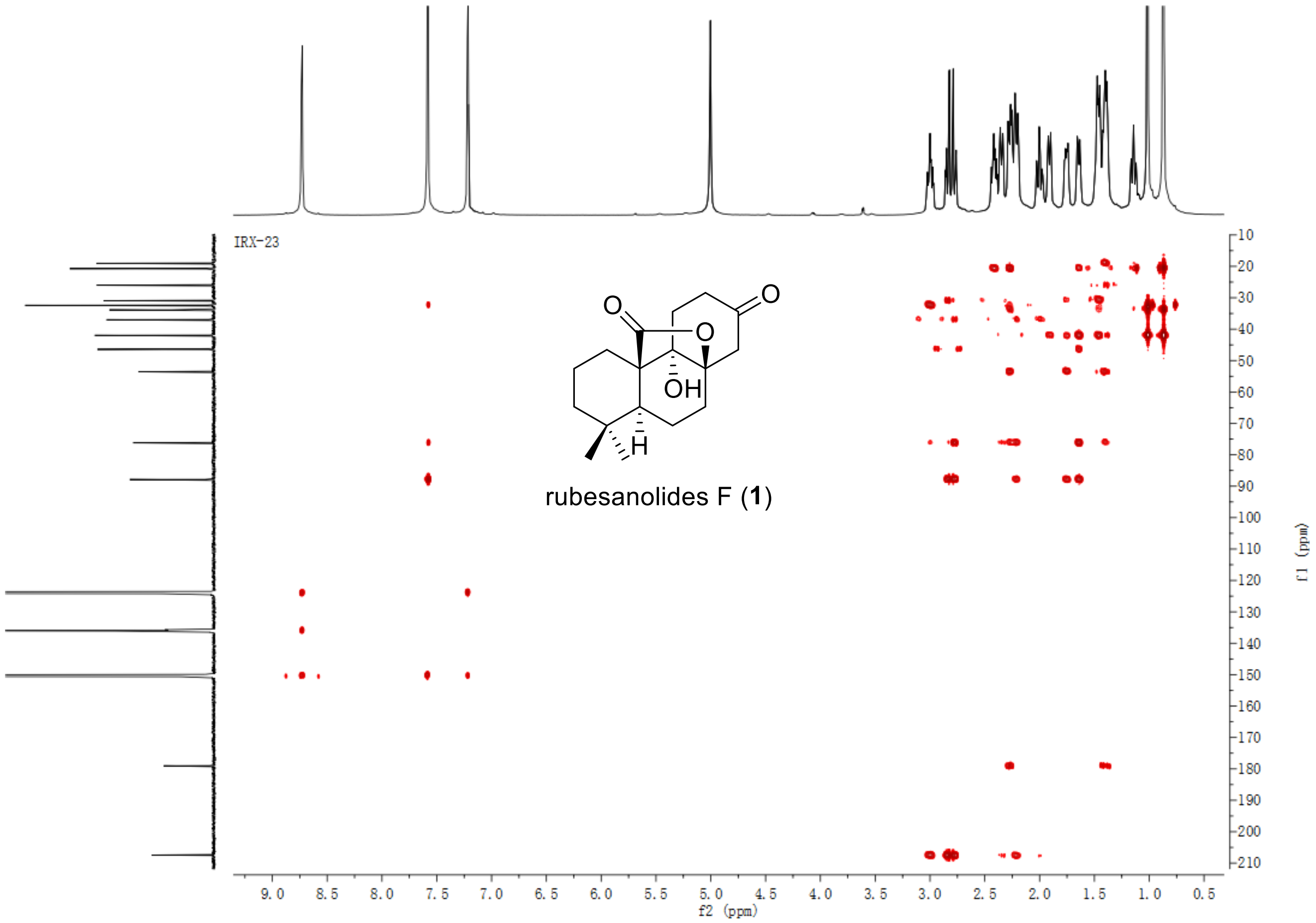

Spectra 4. HMBC spectrum of rubesanolide F (**1**)

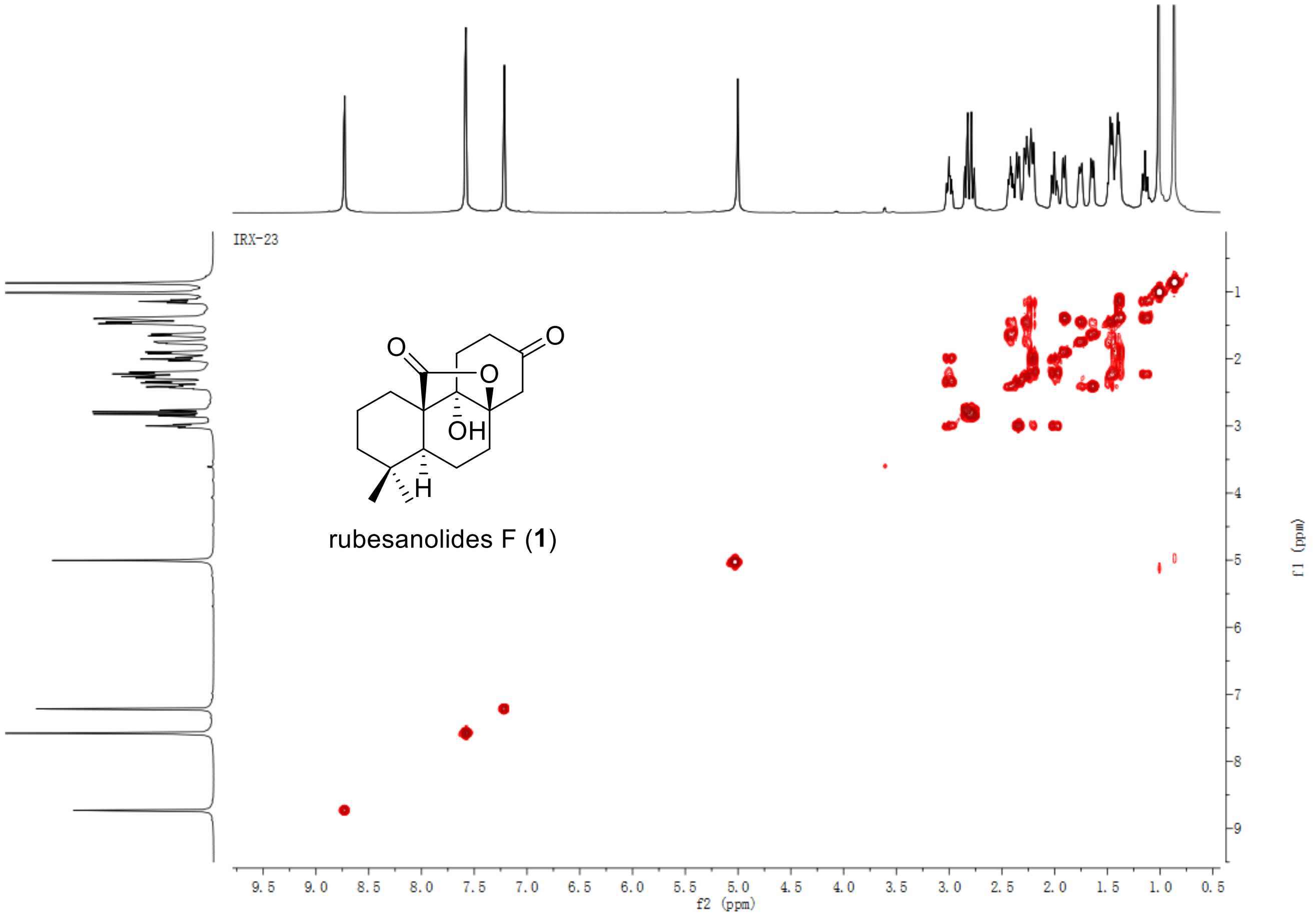

Spectra 5. COSY spectrum of rubesanolide F (**1**)

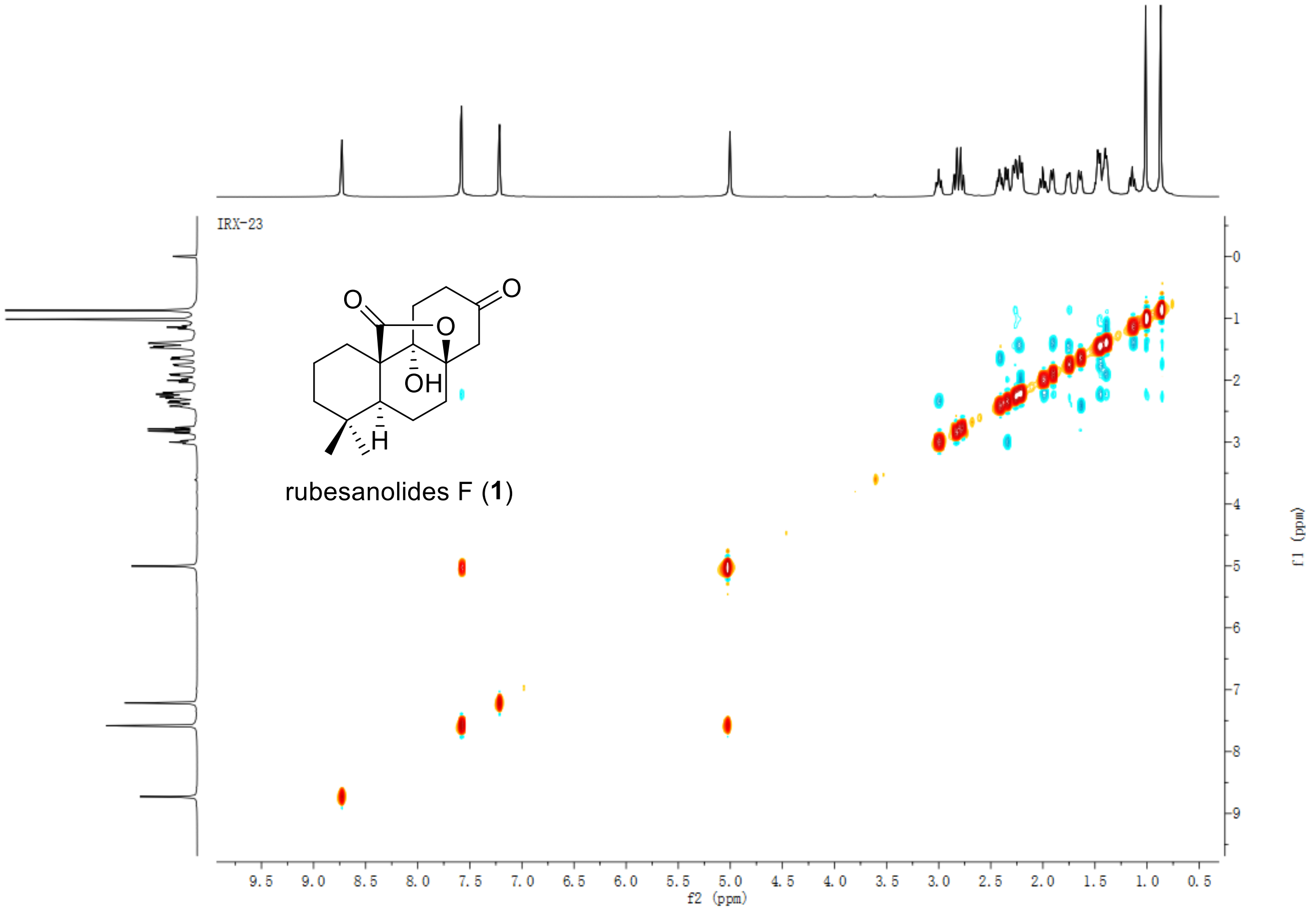

Spectra 6. ROESY spectrum of rubesanolide F (**1**)

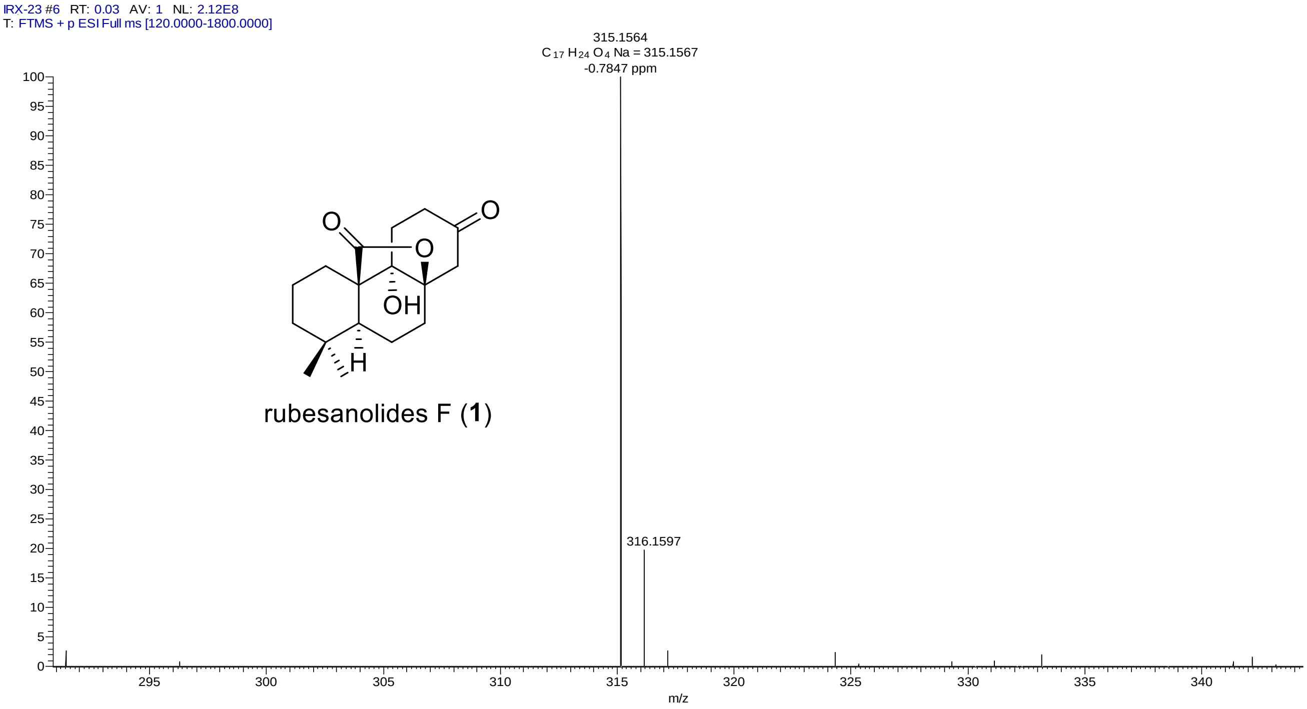

Spectra 7. HRESIMS spectrum of rubesanolide F (**1**)

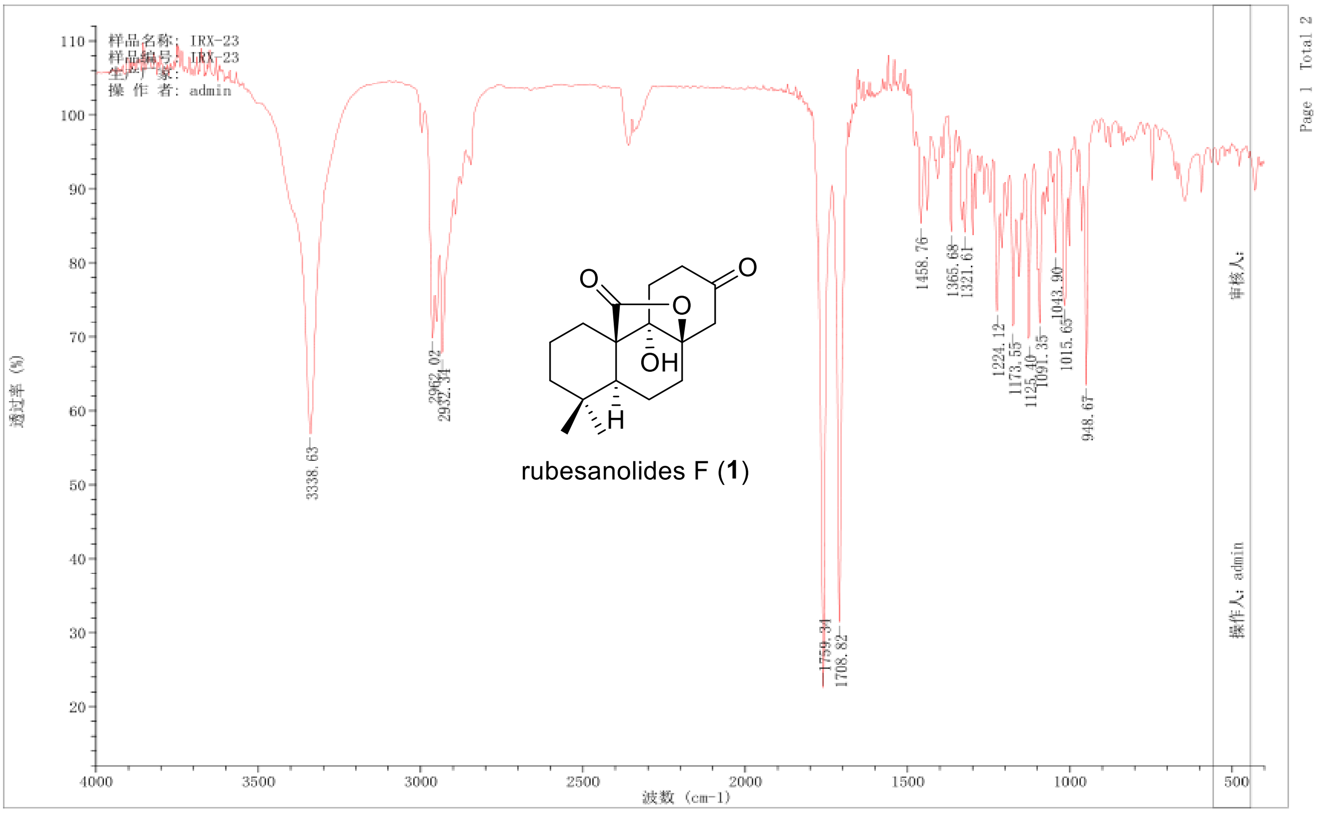

Spectra 8. The IR spectrum of rubesanolides F (**1**)

For rubesanolide G (**2**)

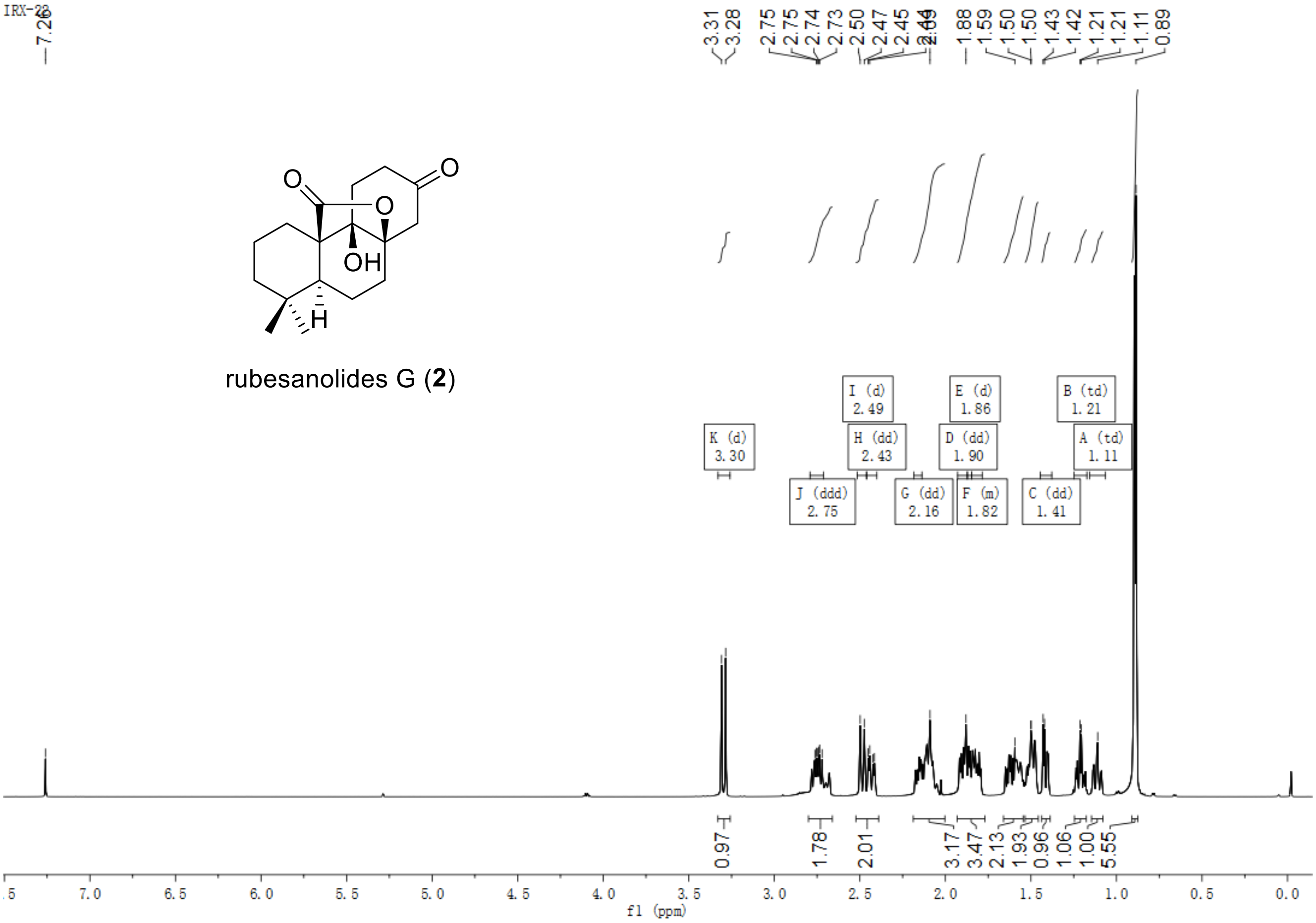

Spectra 9 ^{1}H-NMR spectrum of rubesanolide G (**2**)

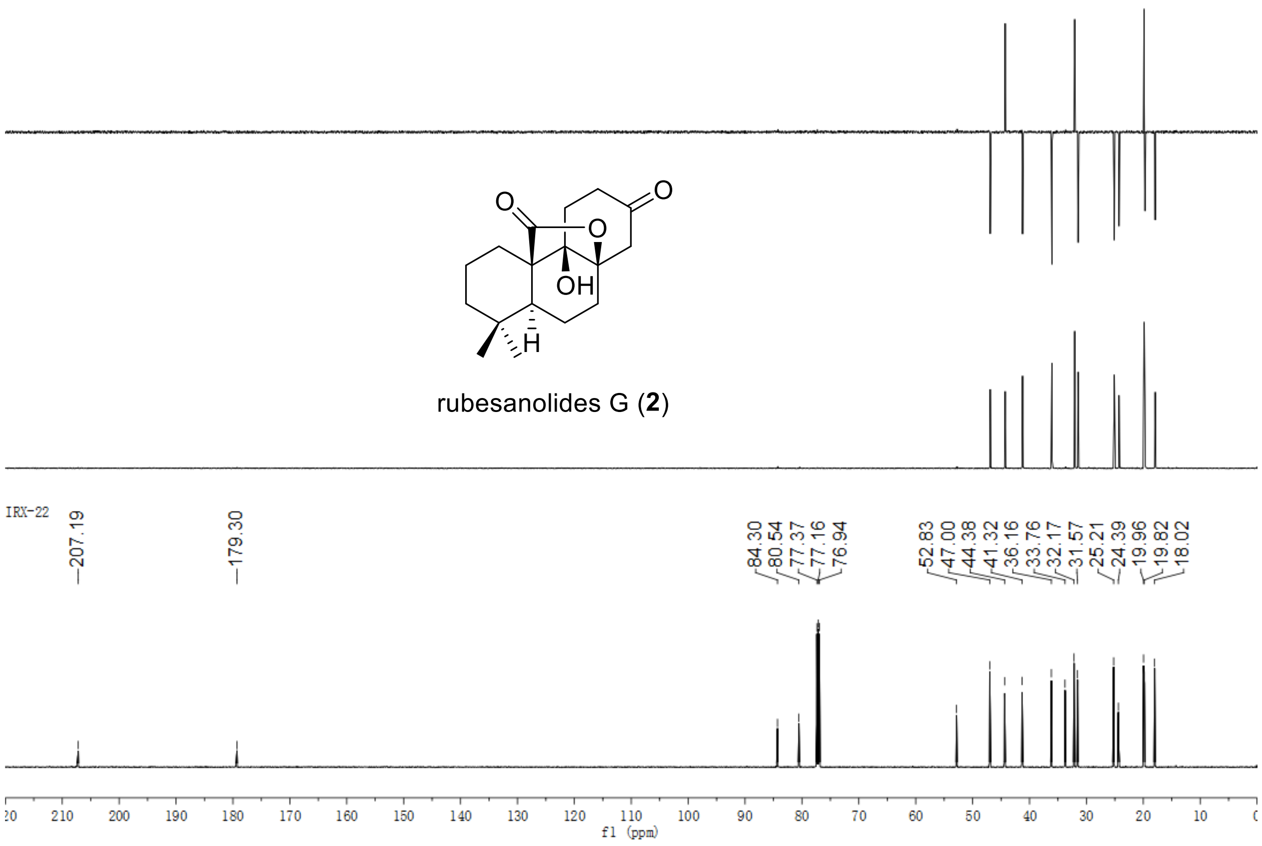

Spectra 10. ^{13}C-NMR and DEPT spectra of rubesanolide G (**2**)

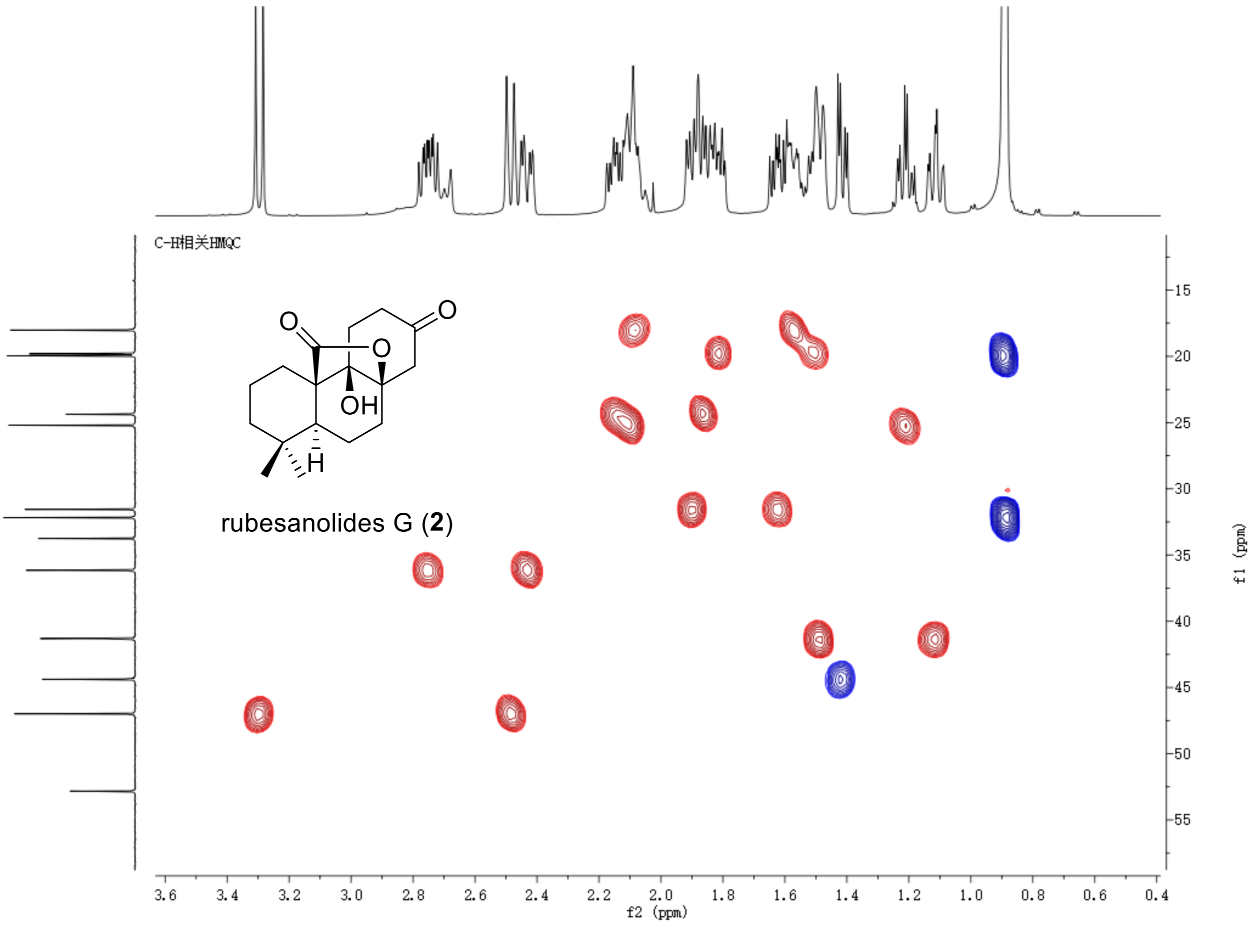

Spectra 11. HSQC spectrum of rubesanolide G (**2**)

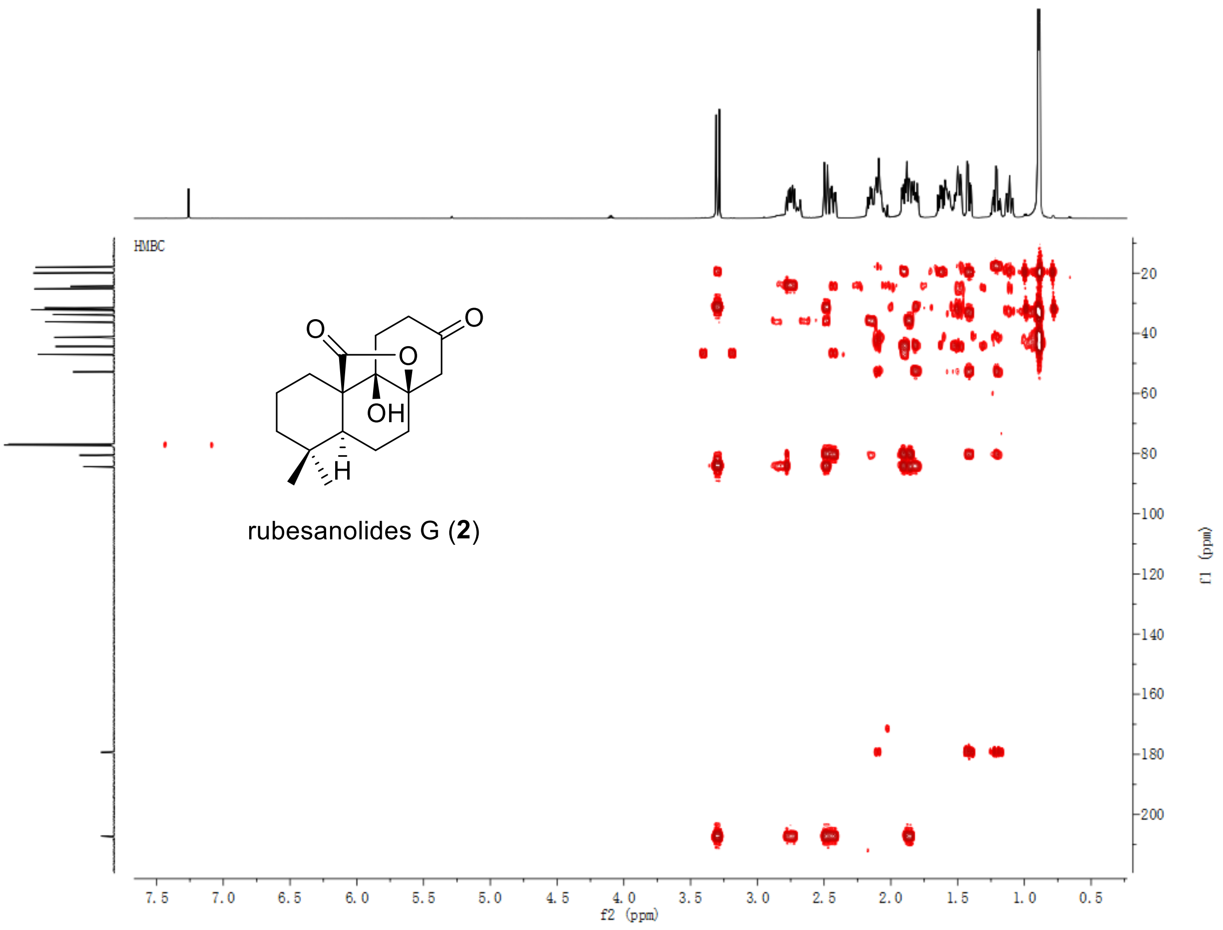

Spectra 12. HMBC spectrum of rubesanolide G (**2**)

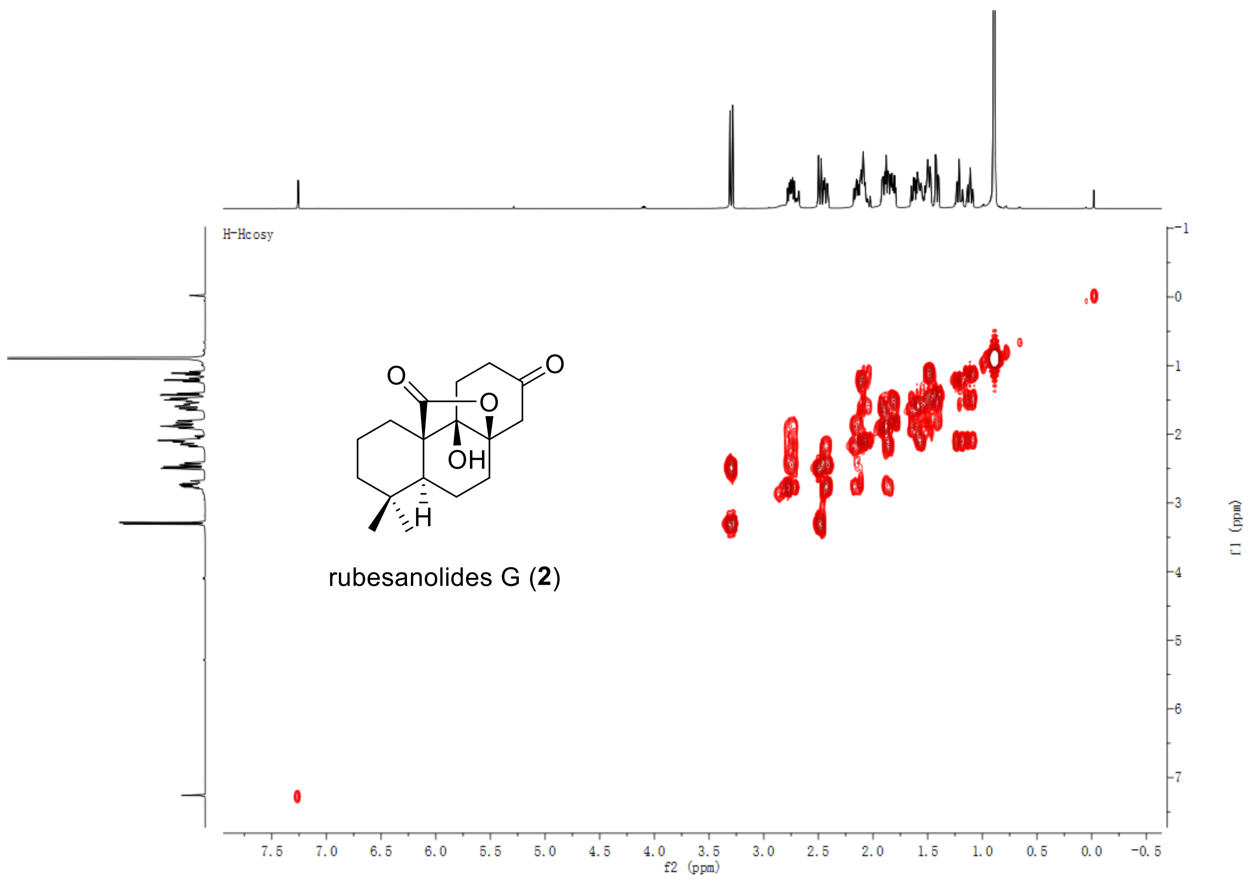

Spectra 13. COSY spectrum of rubesanolide G (**2**)

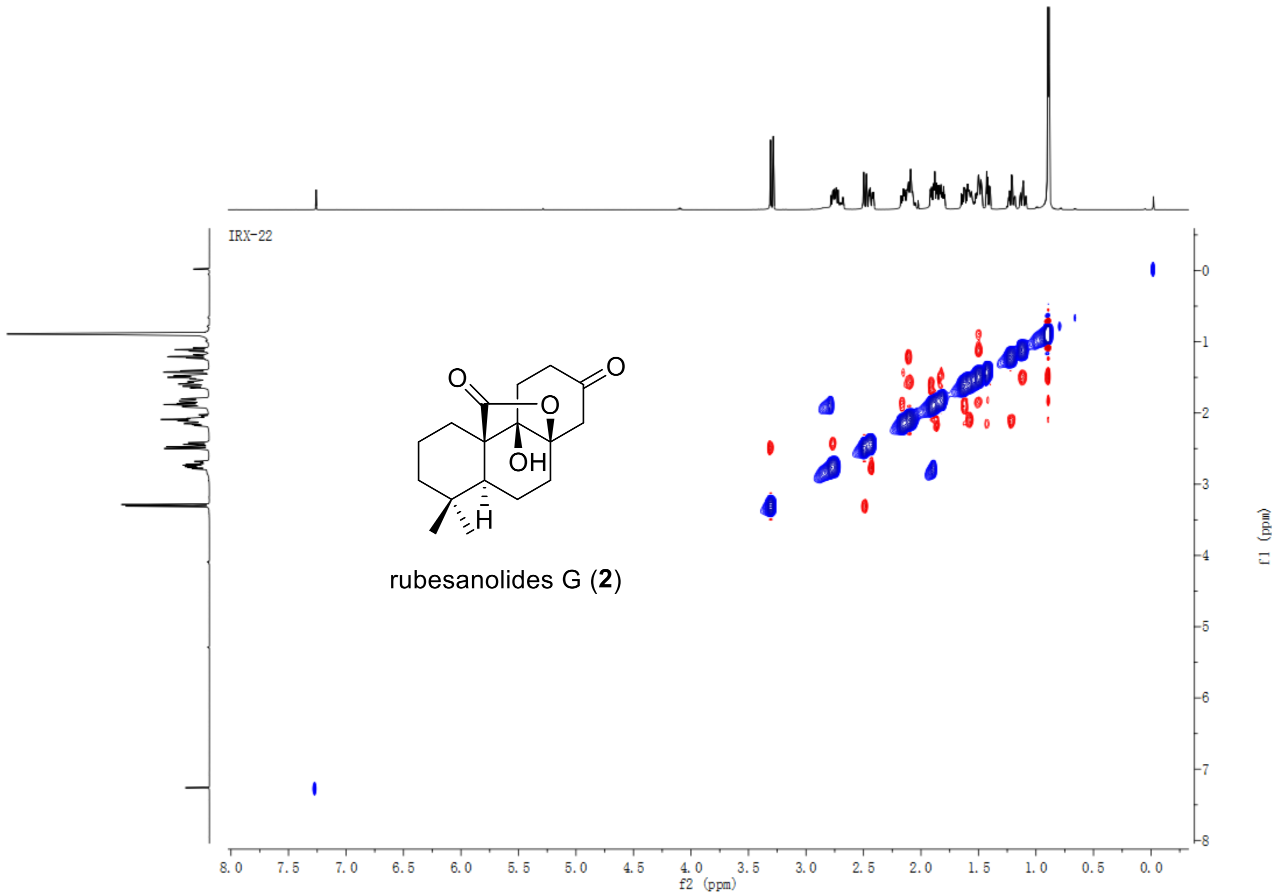

Spectra 14. ROESY spectrum of rubesanolide G (**2**)

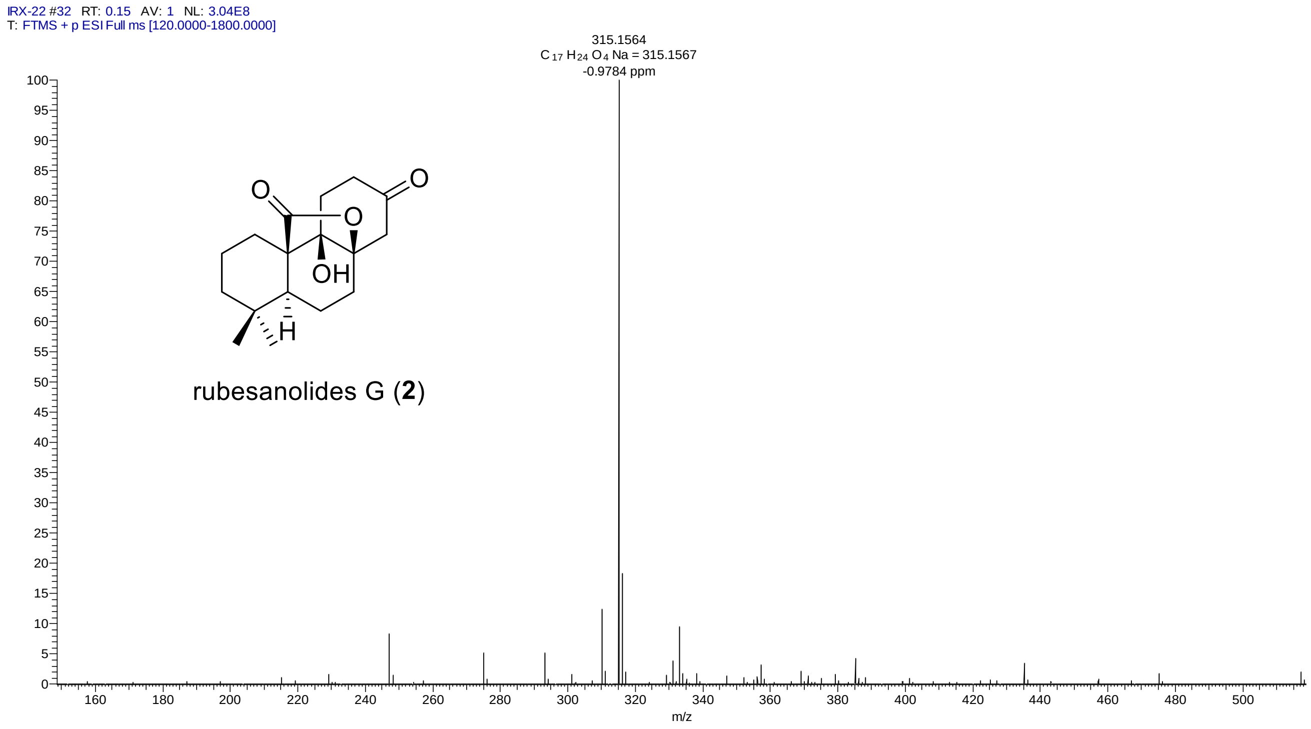

Spectra 15. HRESIMS data of rubesanolide G (**2**)

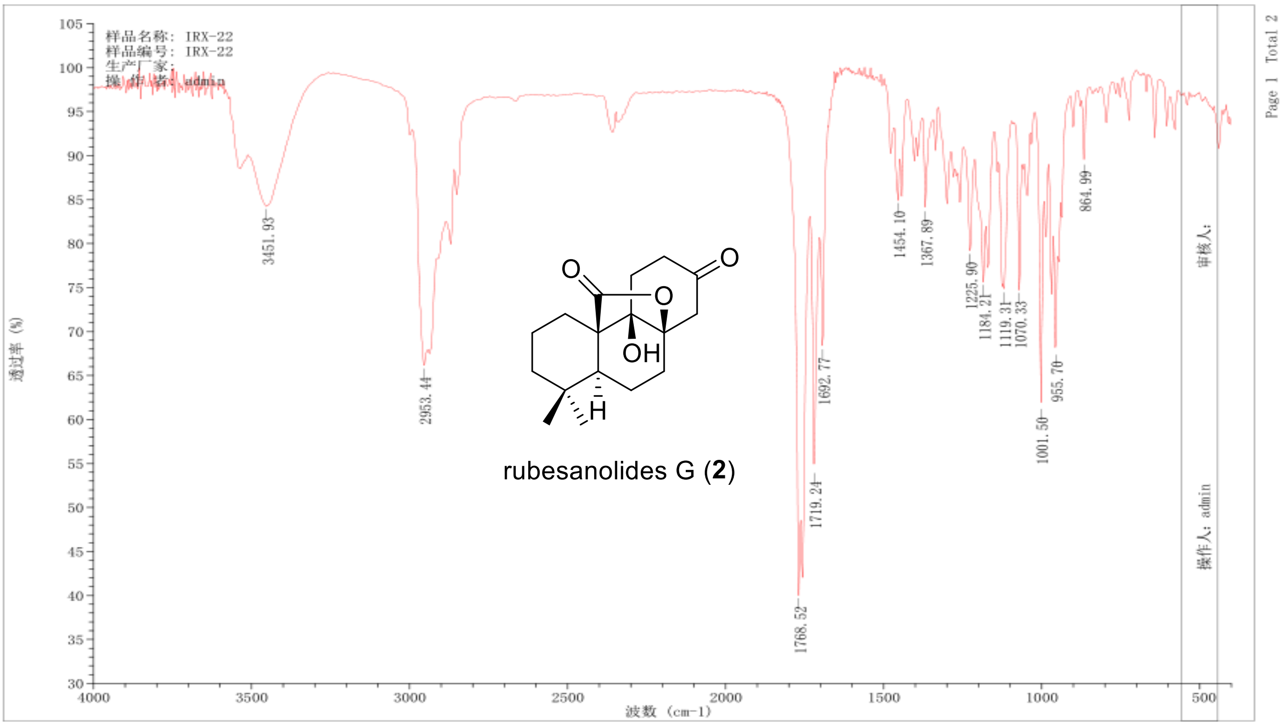

Spectra 16. The IR spectrum of rubesanolide G (**2**)
